# Giant right coronary artery aneurysm with associated dissection: diagnosis and surgical management

**DOI:** 10.1093/jscr/rjaf570

**Published:** 2025-07-25

**Authors:** Henri Bartolozzi, Darragh Rice, Mikaela Forde, Fabio Bartolozzi

**Affiliations:** Department of Cardiothoracic Surgery, University Hospital Galway, Newcastle Rd, Galway, H91YR71, Ireland; Faculty of Medicine, Trakia University, Armeyska St 11, Stara Zagora, 6000, Bulgaria; Department of Cardiothoracic Surgery, University Hospital Galway, Newcastle Rd, Galway, H91YR71, Ireland; Department of Cardiothoracic Surgery, University Hospital Galway, Newcastle Rd, Galway, H91YR71, Ireland; Department of Cardiothoracic Surgery, University Hospital Galway, Newcastle Rd, Galway, H91YR71, Ireland

**Keywords:** giant coronary artery aneurysm, right coronary aneurysm, coronary dissection, angina, syncope, coronary artery bypass graft

## Abstract

Coronary artery aneurysms are defined as a focal dilatation exceeding 1.5 times the diameter of the adjacent normal segment. They are found in up to 5% of patients undergoing coronary angiography. Giant coronary artery aneurysms (GCAAs), typically defined as >20 mm in diameter, with an incidence of ⁓0.02%. We present the case of an 82-year-old female who underwent coronary artery bypass grafting due to a giant right coronary artery aneurysm measuring 85 mm, identified on preoperative contrast-enhanced computed tomography (CT). The patient exhibited symptoms consistent with pericardial irritation secondary to aneurysms size and was found to have significant right heart compression. Surgical intervention included opening and resection of the aneurysmal sac and bypassing the affected segment using a saphenous vein graft. Intraoperatively, it was discovered that the aneurysm wall was dissected, with a high risk of rupture. This case highlights the importance of timely recognition and surgical management of GCAAs.

## Introduction

A focal expansion of the coronary artery that is ˃1.5 times the diameter of the adjacent normal section is known as a coronary artery aneurysm (CAA) [1]. CAAs are a rare clinical entity, occurring most commonly as a sequelae of atherosclerosis, but can be associated with certain disorders such as Kawasaki’s disease. Similar to other aneurysms, they can be classified as saccular or fusiform and although most patients are asymptomatic, they can result in thrombosis, distal embolization, external compression or rupture [2].

Rarely, CAAs can grow to such a size that they are referred to as giant CAAs; their incidence has been reported to be 0.02% [1]. There is no widely recognized, single definition of a giant CAA, In the medical literature, sizes ˃20 mm, 40 mm, 50 mm, and quadruple the reference-vessel diameter have all been suggested as definitive [1]. Location wise, the right coronary artery is the most frequently affected in 40.4% of cases, followed by the left anterior descending artery with 32.3%, with the left main stem and circumflex being rarely involved (3.5%) [3].

## Case presentation

**Figure 1:**
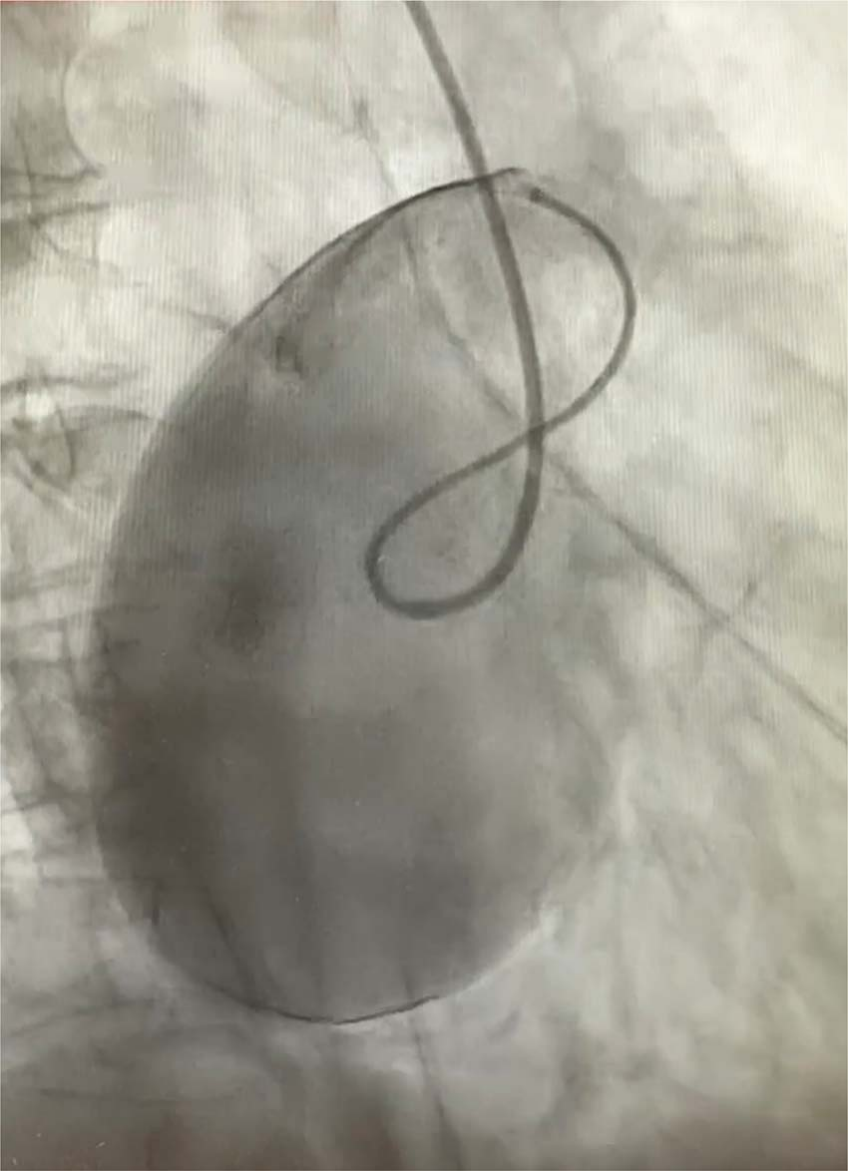
Right coronary angiogram highlighting the aneurysm.

**Figure 2:**
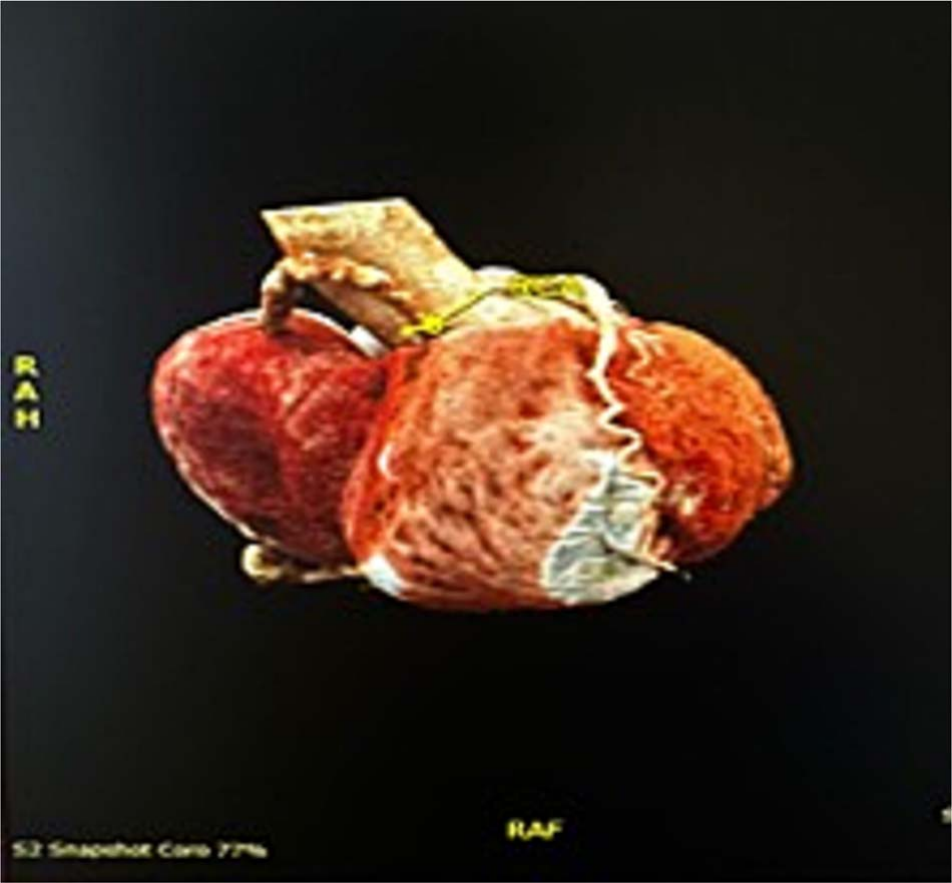
3D reconstruction from CT thorax showing the right coronary aneurysm compressing right heart.

**Figure 3:**
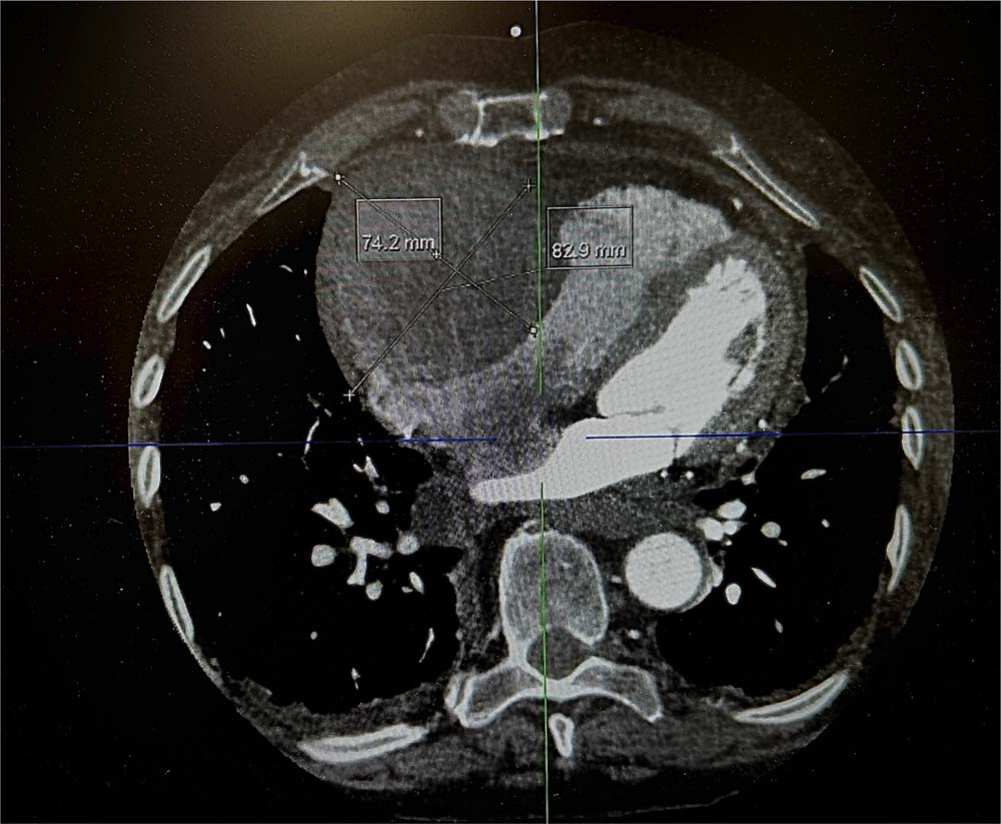
Axial plane of a contrast thorax CT scan showing the giant right coronary aneurysm.

**Figure 4:**
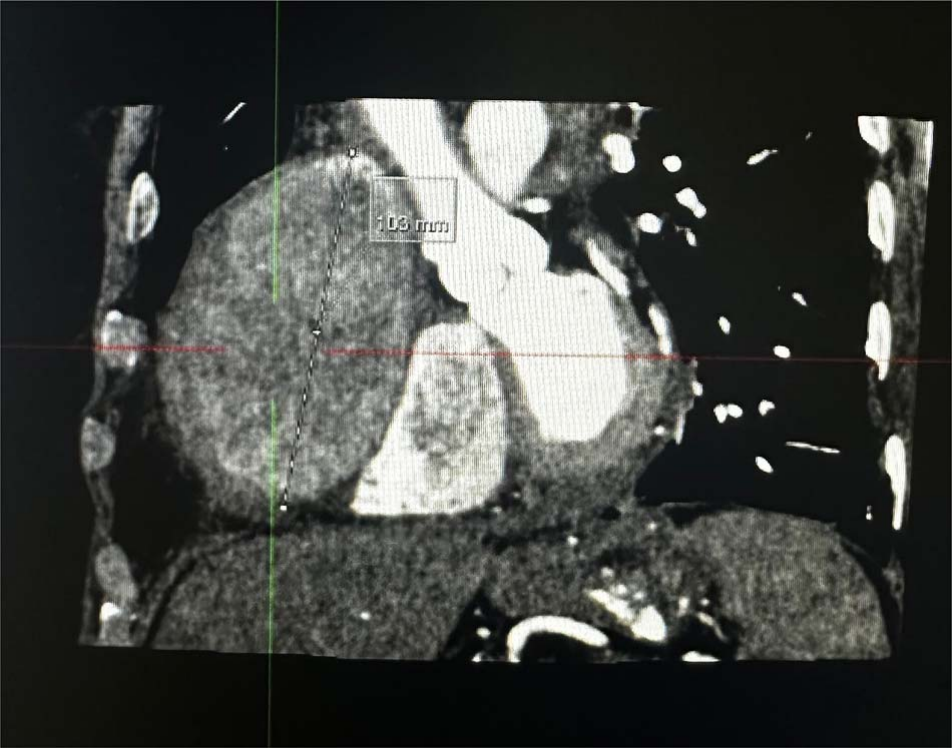
Coronal plane of a contrast thorax CT scan showing the giant coronary aneurysm.

An 82-year-old female presented to the emergency department complaining of progressive severe central chest that was pleuritic in nature, radiating to her right arm. Following a bedside echo, the patient underwent coronary angiography, where a giant right CAA was diagnosed (Fig. 1). Subsequent, contrast computed tomography (CT) scan further characterized the aneurysm, and highlighted significant compression was uncovered compressing both the right atrium and ventricle (Fig. 2). The right coronary aneurysm was measured cross-sectional dimensions of 8.3 × 7.4 cm (Fig. 3) and 10 cm in length (Fig. 4). Following Heart Team discussion, the patient was scheduled for an aneurysmectomy and coronary artery bypass to repair the extensive disease.

**Figure 5:**
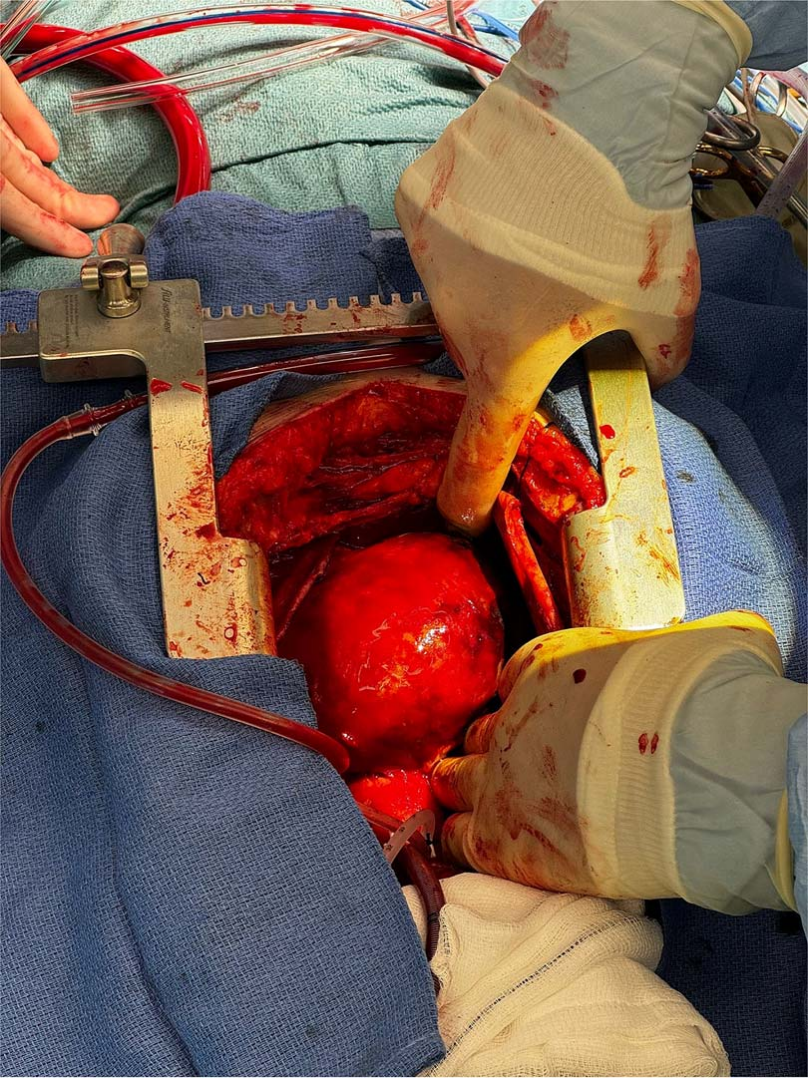
Exposed right coronary aneurysm post sternotomy.

**Figure 6:**
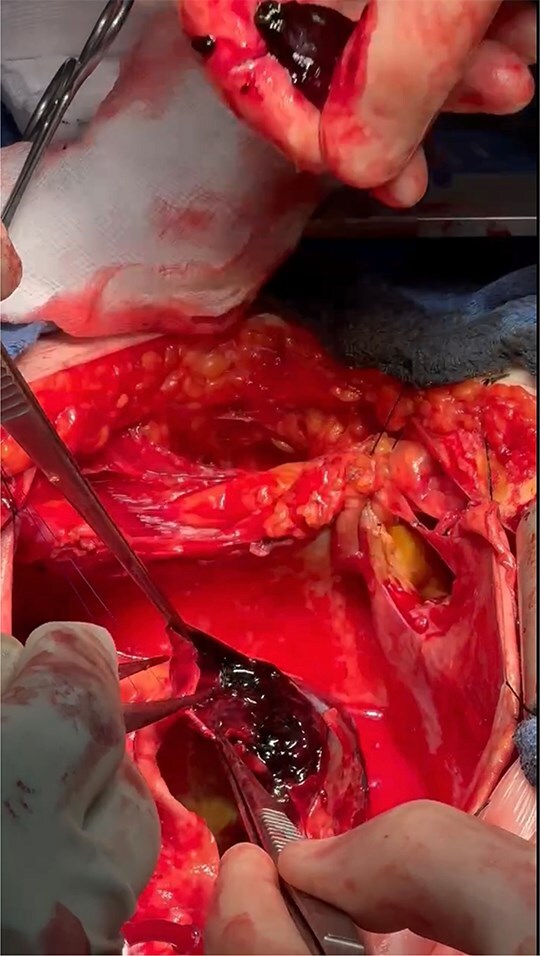
Dissected aneurysm wall with thrombus formation.

**Figure 7:**
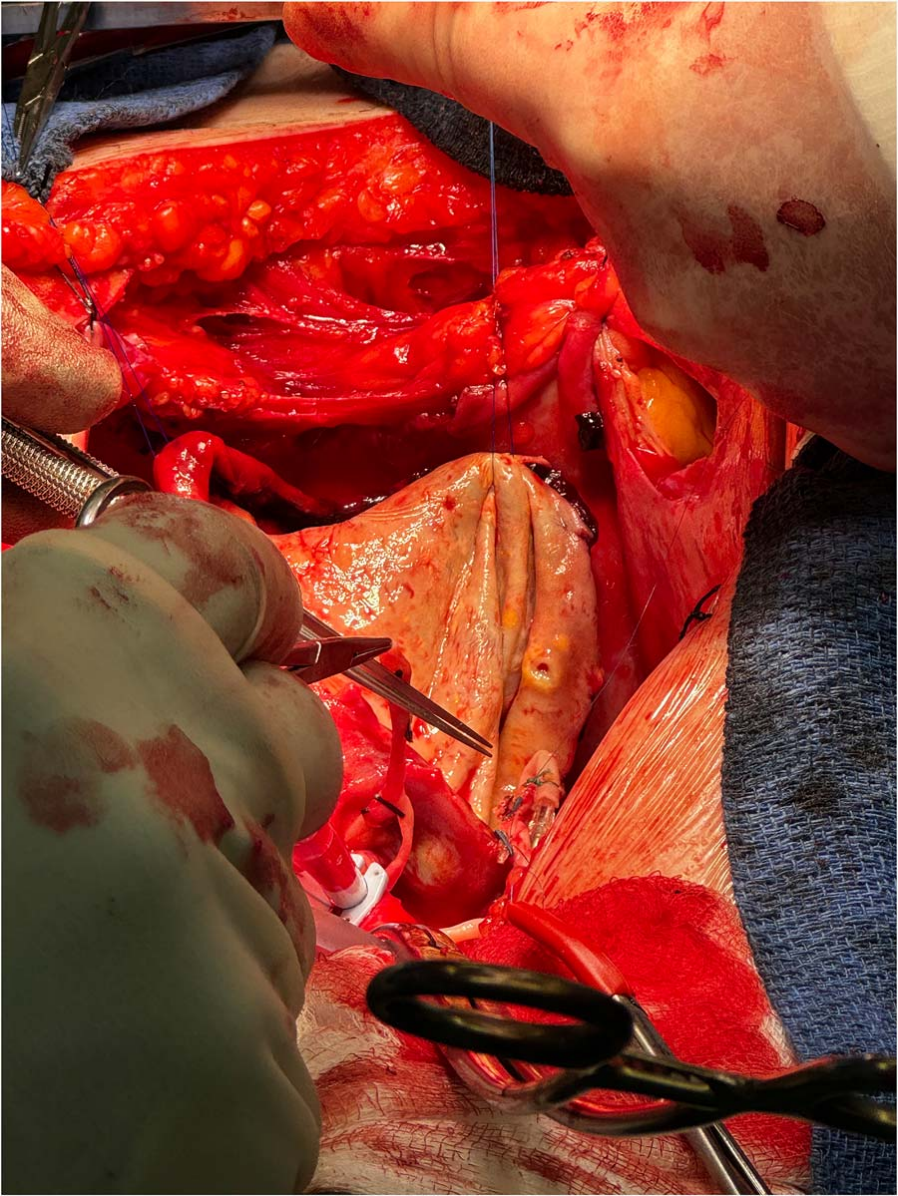
Direct anastomosis of saphenous conduit to the distal neck of the aneurysm using an end-to-end technique.

The operation was performed via median sternotomy, with the aneurysm immediately visible post sternotomy (Fig. 5). The pericardium was carefully opened without complication, and due to the right atrium being inaccessible, cardio-pulmonary bypass was established using central ascending aortic cannulation and femoral venous cannulation, with antegrade cardioplegia (Fig. 5). The giant CAA was then opened directly where it was uncovered that the aneurysm wall had also dissected, and was paper thin (Fig. 6). The thrombus was evacuated, perforating branches were identified and oversewn, of which there were two. Following this, an endoscopically harvested saphenous vein graft was used to directly, end-to-end anastomose the proximal and distal necks of the aneurysm, bypassing the sac entirely (Fig. 7). The patient was successfully weaned from Cardiopulmonary Bypass and the procedure concluded without complication.

**Figure 8:**
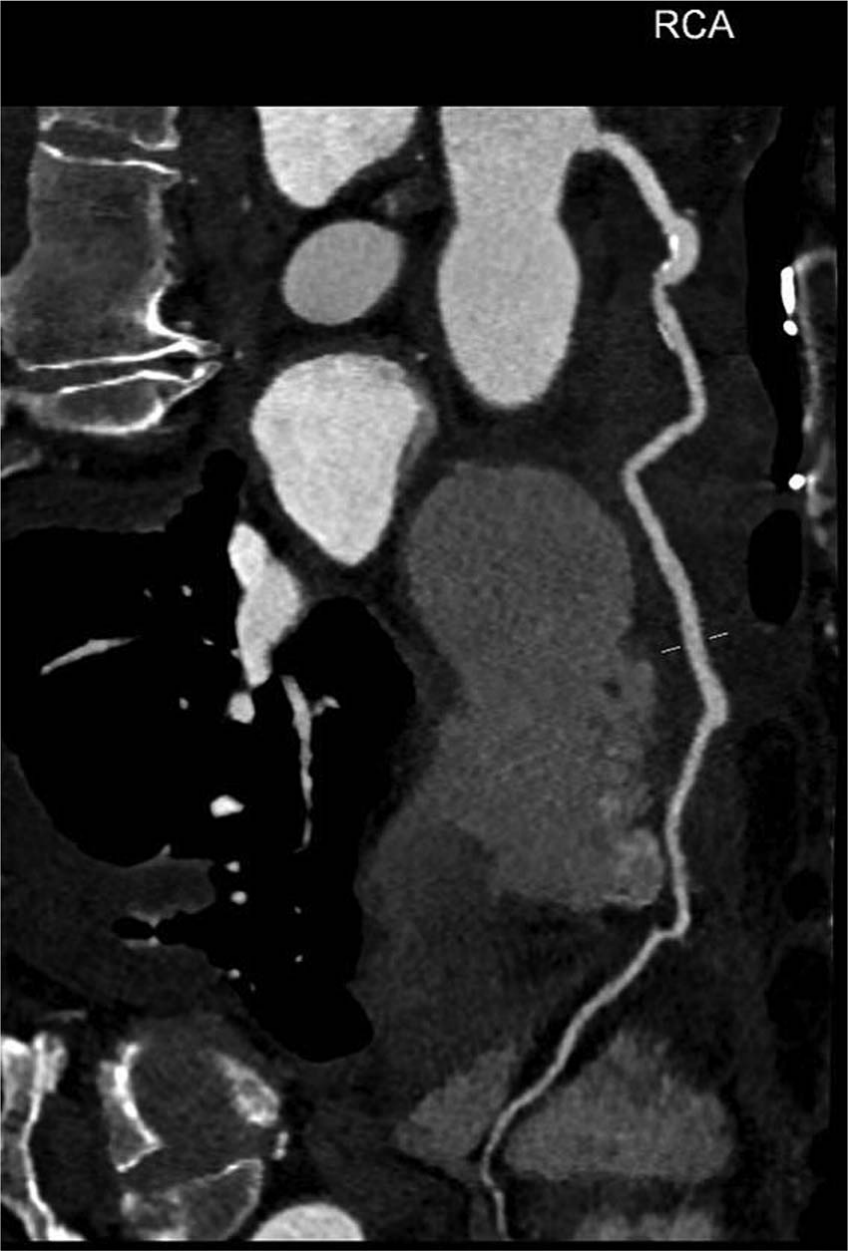
Reconstructed path of right coronary artery including vein graft via aneurysmal sac from post-operative CT coronary angiogram.

Following the surgery, the patient remained in intensive care for 2 days, and progressed as expected over the coming week. She was discharged on day 8 postoperatively, with the only postoperative complication being a short run of an atrial tachycardia that was medically managed and was in sinus rhythm at the time of discharge. Prior to discharge a CT Coronary Angiogram with contrast was performed which highlights the repair (Fig. 8). She was reviewed in outpatients six weeks post procedure and has made a full recovery.

## Discussion

The prevalence of CAAs on angiography ranges from 0.15% to 4.9%, making them extremely uncommon, with a higher prevalence in men [4]. However, giant coronary aneurysms are even more rare with an incidence of 0.02%. In adults, atherosclerosis is the cause of half of CAAs making it the most prevalent cause of these conditions [5]. Kawasaki disease, vasculitidies (polyarteritis nodosa and Takayasu's arteritis), lupus erythematosus, connective tissue disorders, congenital defects, infections (like Lyme disease, syphilis, and narcotic emboli), trauma, dissection, cocaine abuse, and iatrogenic and idiopathic origins, genetic causes such as connective tissue diseases like Marfan's and Ehlers-Danlos syndromes are further known causes [3, 6, 7].

In patients with CAAs, surgical management becomes necessary when aneurysms are symptomatic or large, as these can lead to serious complications like rupture or myocardial ischemia. Surgical approaches generally involve aneurysm resection or ligation, frequently accompanied by coronary artery bypass grafting to restore adequate blood flow when the aneurysm obstructs a major artery. In more challenging cases, involving critical coronary branches, advanced grafting techniques are necessary to optimize perfusion and prevent ischemic events [8]. Although surgery carries a high level of risk, it remains essential in preventing potentially severe outcomes associated with untreated CAAs [8].

## Conclusion

This case illustrates the importance of early diagnosis and tailored surgical management for giant CAAs, which can lead to severe complications like cardiac compression and rupture. Prompt imaging and surgical intervention, including aneurysm resection and bypass grafting, enabled a positive outcome for this patient.
